# Influence of relative humidity in *Vibrio cholerae* infection: A time series model

**Published:** 2011-02

**Authors:** K. Rajendran, A. Sumi, M.K. Bhattachariya, B. Manna, D. Sur, N. Kobayashi, T. Ramamurthy

**Affiliations:** *National Institute of Cholera & Enteric Diseases, Kolkata, India*; * *Department of Hygiene, Sapporo Medical University, Japan*

**Keywords:** Cholera, GLM, relative humidity, SARIMA, temperature, rainfall, *V. cholerae*

## Abstract

**Background & objectives:**

Spread of cholera in West Bengal is known to be related to its ecosystem which favours *Vibrio cholerae*. Incidence of cholera has not been correlated with temperature, relative humidity and rainfall, which may act as favourable factors. The aim of this study was to investigate the relational impact of climate changes on cholera.

**Methods:**

Monthly *V. cholerae* infection data for of the past 13 years (1996-2008), average relative humidity (RH), temperature and rainfall in Kolkata were considered for the time series analysis of Seasonal Auto-Regressive Integrated Moving Average (SARIMA) model to investigate relational impact of climatic association of *V. cholerae* infection and General Linear Model (GLM) for point estimation.

**Results:**

The SARIMA (1,0,0)(0,1,1) model revealed that monthly average RH was consistently linear related to *V. cholerae* infection during monsoon season as well as temperature and rainfall were non-stationary, AR(1), SMA(1) and SI(1) (*P*<0.001) were highly significant with seasonal difference. The GLM has identified that consistent (<10%) range of RH (86.78 ± 4.13, CV=5.0, *P* <0.001) with moderate to highest (>7 cm) rainfall (10.1 ± 5.1, CV=50.1, *P* <0.001) and wide (>5-10°C) range of temperature (29.00 ± 1.64, CV=5.6, *P* <0.001) collectively acted as an ideal climatic condition for *V. cholerae* infection. Increase of RH to 21 per cent influenced an unusual *V. cholerae* infection in December 2008 compared to previous years.

**Interpretation & conclusions:**

*V. cholerae* infection was associated higher RH (>80%) with 29°C temperature with intermittent average (10 cm) rainfall. This model also identified periodicity and seasonal patterns of cholera in Kolkata. Heavy rainfall indirectly influenced the *V. cholerae* infection, whereas no correlation was found with high temperature.

Global disease burden studies published in 2008 showed that the diarrhoeal disease alone was responsible for a loss of 72.8 million DALYS with mortality rate of about 2.2 million per year^1^. In most of the cases (88%), the diarrhoea is attributed to the unsafe water supply and inadequate sanitation^2^. Cholera is one of the dreadful diseases with pandemic diffusion caused by *Vibrio cholerae* O1 and O139 serogroups^3^. Seven distinct pandemics of cholera have been reported including the one caused by the novel serogroup O139 in the year 1993^4^. Many studies carried out in the past have shown that cholera appears in the form of outbreaks. Recently, cholera was reported in Angola with 1437 deaths out of 38897 cases and the case fatality rate (CFR) was 3.6 per cent^5^. Prevention of the spread of infectious diseases requires early detection, prediction and early warning of outbreaks or large scale infections.

Infectious disease transmission should be viewed with certain ecological factors which vary from region to region. Cholera transmission is mainly related to the environmental factors which are directly related to climatic variability. In developing countries, the environment is deteriorating fast due to explosive increase of human population. Previous studies have shown that unhygienic and socio-economic factors were related to the diarrhoeal outbreaks in West Bengal, India^6^.

The seasonal changes in West Bengal have been studied over six decades and the patterns of cholera have also changed consequently. *V. cholerae* infection was higher in April and May, but has been shifted recently to September and October with two peaks annually^7^,^8^. Spread of cholera in West Bengal is related to its ecosystem and its physical environment which favours *V. cholerae*^7^,^8^. However, the incidence of cholera has not been correlated well with many conducive factors such as temperature, relative humidity and rainfall.

In health statistics, timely prediction is very important to prevent health hazards to the community. Emergence of several viral diarrhoea infections due to climate variability in Bangladesh, Thailand, Australian cities and China have been reported^9^–^14^ but a few studies were made on cholera with time series model^15^–^18^. In this study, an attempt was made to find the influence of season on cholera using time Series Analysis and generalized linear model with special emphasis on relational impact and climate factors.

## Material & Methods

In this study, data were collected over thirteen years (January 1996 to December 2008) from an active diarrhoeal surveillance system at the Infectious Diseases Hospital (IDH), Kolkata. In the surveillance, every 5^th^ hospitalized patient in two randomly selected days in a week was enrolled. Date on local relative humidity, temperature and rainfall were collected from Meteorological Department, Kolkata. The hospital based active surveillance data were considered to determine the patterns of the *V. cholerae* infection among the admitted patients using the time series model and generalized linear model (GLM) for point prediction^19^,^20^. Month-wise data were structured for *V. cholerae* (*V. cholerae* O1, O139 and non-O1, non-O139) positive cases along with mean monthly RH temperature and rainfall for seasonal auto-regressive integrated moving average (SARIMA) model, daily *V. cholerae* (inclusive of O1, O139 and non-O1, non-O139 serogroups) positive cases and RH, temperature and rain fall for GLM. The data were checked to exclude missing information and plotted in a sequential curve.

In climatic factors, the difference of RH and temperature [*i.e*., morning (max)-evening (min)] were used in the analysis. This procedure was relevant to identify the actual causative factors instead of mean factors. The mean factors purposefully have been averted to avoid the influence of high variation in the series. Since the sequential curve showed upward trend and distinct seasonal pattern in hospital admitted patients at the IDH, an exponential smoothing with simple, hot and winter models was used after the data differentiated to derive stationary because the data were non-stationary to identify the model.

The existing seasonal pattern permitted to grow with the upward series trend that suggested multiplicative seasonality. The winter model was checked by sequential chart with fitted values and *V. cholerae* infections. Among the three models, different assumptions on trend and seasonality have been evidenced by the winter model and explored good visual fit to the data.

The model adopted in this study presented trend and seasonality with set of predictor variables for RH, temperature and rainfall. A stationary time series has a mean and variance that are essentially constants through time. This series was non-stationary, as the ARIMA technique differentiates a non-stationary series one or more times until the resulting series is stationary. The SARIMA model is a special case of non-stationary data and auto regression (AR) polynomials are constrained to equal unity^21^.

To ensure the variability of relative humidity in relation to *V. cholerae* infection, the data were divided into rainfall or no rainfall with *V. cholerae* infection. Employing GLM with linear scale response, the number of daily *V. cholerae* infection was categorized as favourable interval scale of temperature and RH. The model effects were considered foremost to intercept in the model with hybrid fisher scoring to get the maximum likelihood estimation. The model effect analysis was type III with 95 per cent confidence interval, likelihood ratio, chi-square statistics and its profile likelihood confidence interval to know the model information and to elevate the favourable factors such as RH, temperature and rainfall ranges as well as the seasons by GLM. Among these factors, the rainfall was used as a co-variant to know the influence of the other factors.

## Results

The outcome variables have been created by difference of morning and evening observed temperatures and relative humidity and converted as seven categorized factors for RH and four factors for temperature. This procedure was efficient and accurate evaluation than the mean values for climatic factors to estimate the predictive ranges of RH and temperature by GLM.

The difference of RH (%) was categorized into seven factors (1= highest days evening, 2=zero difference days, 3= <10%, 4= >10-20%, 5= >20-30%, 6= >30-40% and 7= >40%). The temperature (°C) difference was categorized in to 4 factors (1= <5°C, 2= >5-10°C, 3= >10-15°C and 4=>15°C) and the seasonality was categorized into pre monsoon (March-May), monsoon (June-September), post monsoon (October-November) and winter (December-February).

### 

#### Primary analysis:

In a total of 2544 surveillance days over a period of 13 years, 2719 *V. cholerae* infected (culture positive) cases were recorded in 1226 days. In the RH outcome variable, the highest evening RH was in 489 days, of which 302 (61.7%) days had 713 *V. cholerae* cases (2.4 cases/day), the zero difference days were 95, of which 57 (60%) days had 144 *V. cholerae* cases (2.5 cases/day), ≤10 per cent difference days were 983, of which 525 (53%) days had 1180 *V. cholerae* cases (2.2 cases/day), >10-20 per cent difference days were 605, of which 250 (41%) days had 537 *V. cholerae* cases (2.1 cases/day), >20-30 per cent difference days were 257, of which 71 (27.6%) days had 107 *V. cholerae* cases (1.5 cases/day), >30-40 per cent difference days were 94, of which 17 (18%) days had 27 *V. cholerae* cases (1.6 cases/day) and >40 per cent difference days were 21, of which 4 (19%) days had 5 *V. cholerae* cases (1.2 cases/day).

In the temperature outcome variable, <5°C difference days were 290, of which 189 (65.2%) days had 433 *V. cholerae* cases (2.3 cases/per day), >5-10°C difference days were 1367, of which 780 (57%) days had 1836 *V. cholerae* cases (2.3 cases/per day), >10-15°C difference days were 858, of which 251 (29.2%) days had 439 *V. cholerae* cases (1.7 cases/per day) and >15°C difference days were 29, of which 6 (20.7%) days had 11 *V. cholerae* cases (1.8 cases/per day).

Among seasons, 642 days were pre monsoon days, of which 276 (43.0%) days had 483 *V. cholerae* cases (1.7 cases/per day), monsoon had 864 days, of which 578 (66.9%) days had 1397 *V. cholerae* cases (2.4 cases/per day), post monsoon had 431 days, of which 286 (65.4%) days had 719 *V. cholerae* cases (2.5 cases/per day) and the winter had 607days, of which 86 (14.2%) days had 120 *V. cholerae* cases (1.4 cases/per day).

#### SARIMA model:

This model was explored with a special approach in identifying seasonal orders of Auto correlation Factor (ACF) and Partial Auto correlation Factor (PACF) plots at seasonal lags extending up to lag 48. The PACF showed insignificant in seasonal order and the ACF plot showed spike in lag 12 without strong evidence of a substantial tail. The SAR (1) model found to be unsuitable in this study. Apart from the model that had combination of SI (1) and SMA (1) in seasonal model and the other models were avoided. The SMA (1) model was fitted in the series as there was an exponential declining in ACF of SARIMA.

With SARIMA (1, 0, 0) (0, 1, 1) of ACF and PACF, the spike of 1 ^st^ lag explored that the requirement of inclusion of AR (1) on non-seasonal models was to stabilize the model. The created candidate model of *V. cholerae* with predictor variables of RH, temperature and rainfall were mixed in SARIMA (1, 0, 0) (0, 1, 1) along with seasonal difference to stabilize model. The periodicity, seasonality and pattern of *V. cholerae* infection were investigated for future prevention. Heavy rain fall indirectly stimulated the *V. cholerae* infection. High RH favoured *V. cholerae* infection, that was linearly related whereas high temperature (mean) did not favour the *V. cholerae* infection (Table I).

**Table I:** Identified ARIMA (1,0,0) (0,1,1) Model depicts climatic impact for rain fall temperature and relative humidity of the *V. cholerae* infection at IDH, Kolkata

Model/parameter	Estimate	t- value	*P* value
Rainfall: Non seasonal lags			
AR(1)	0.638	9.724	<0.001*
Seasonal lags			
MA(1)	0.809	9.721	<0.001*
I(1)	Seasonal difference		
Temperature: Non seasonal lags			
AR(1)	0.638	9.724	<0.001*
Seasonal lags			
MA(1)	0.809	9.721	<0.001*
I(1)	Seasonal difference		
Relative humidity: Non seasonal lags			
AR(1)	0.645	9.909	<0.001*
Seasonal lags			
MA(1)	0.792	9.721	<0.001*
I(1)	Seasonal difference		
Relative humidity	0.638	2.627	0.010*

* Statistically significant

#### Generalized linear model:

Generalized linear model was employed to explore the intensity of various level of RH as the SARIMA revealed that it had a linear seasonal relation and the temperature and rainfall were non-stationary with significant relation in this model. This model carried the different level of RH percentage in the analysis which showed the highly significant association with *V. cholerae* infection over 13 years.

#### V. cholerae infection with rainfall:

**Fig. 1:**
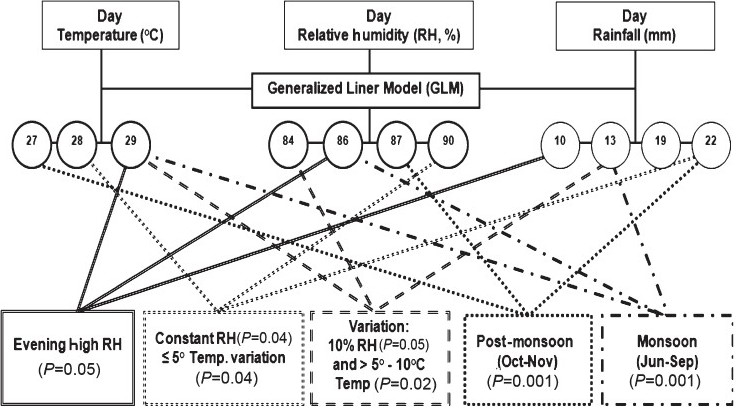
GLM identified *V. cholerae* favourable difference ranges of climatic factors with rainfall (n=598).

Fig. 1 depicts that evening high RH (*P*=0.05) significantly favoured *V. cholerae* infection while RH and temperature were 86 per cent and 29°C, respectively with rainfall around 10 mm. The zero difference RH co-incidence with least variation of day temperature (<5°C) favoured *V. cholerae* infection while RH (*P*=0.04) and temperature (*P*=0.04) were 90 per cent and 28°C respectively with high rainfall (22 mm). The RH variation (*P*>0.05) was 10 per cent in day co-incidence with temperature variation >5-10°C (*P*=0.02) favoured *V. cholerae* infection while RH and temperature were 84 per cent and 29°C respectively with rainfall 13 mm. The monsoon season was the significantly favoured season for *V. cholerae* (*P*<0.001) infection with high RH (86%), moderate temperature (29°C) and minimum average days rainfall (13 mm). Moreover, the post-monsoon season was also significantly supported *V. cholerae* infection (*P*<0.001) with favourable temperature, RH and rainfall (27°C, 86% and 13 mm, respectively; Table II).

**Table II:** Generalized linear model (GLM) explored the *V. cholerae* infection with rain fall events (n=598) associated with interval rang of relative humidity, temperature and rainfall

R.H. Difference (Morning-Evening)	Mean ± SD (Median)	*V. cholerae* (n=589)	Parameter value	SE 95% likelihood CI	Wald Chi-square	*P* value
Evening high RH (n=170)	86.10 ± 2.1Rh	170	0.522	0.28 (.-55-1.59)	3.56	0.059*
	29.05 ± 1.86 T					
	9.76 ± 14.71(3.9)R					
Day’s equal RH (n=35)	90.03 ±} 7.67 Rh	35	1.074	0.52 (-.10-2.26)	4.27	0.039*
	28.38 ±} 2.01 T					
	21.59 ±} 23.78(11.80)R					
≤10% (n=225)	83.96 ±} 7.68 Rh	255	0.485	0.27 (-.57-1.54)	3.17	0.075
	28.58 ±} 1.97 T					
	15.01 ±} 21.19(6.0)R					
>10-20% (n=118)	78.93 ±} 8.83 RH	188	0.466	0.27 (-.61-1.54)	2.96	0.086*
	28.98 ±} 2.24 T					
	13.40 ±} 19.62(4.9)R					
>20-30% (n-11)	69.27 ±} 9.22 Rh	11	Reference category			
	28.71 ±} 2.67 T					
	5.63 ±} 10.48(.60)R					
Temperature difference						
≤5°C (n=172)	27.56 ±} 1.93T	172	0.760	0.38 (-1.65-3.19)	4.00	0.045*
	87.99 ±} 6.24 RH					
	17.64 ±} 21.52(9.80) R					
>5-10°C (n=361)	29.28 ±} 1.87T	361	0.856	0.38 (-1.55-3.26)	5.06	0.024*
	83.43 ±} 7.34 RH					
	11.32 ±} 18.47(3.60) R					
>10-15°C (n=54)	29.46 ±} 1.56T	54	0.210	0.39 (-2.24-2.66)	0.288	0.591
	71.73 ±} 10.47 RH					
	13.47 ±} 16.96(5.30) R					
>15°C (n=2)	27.70 ±} 0.14T	2	Reference category			
	73.00 ±} 9.19 RH					
	19.20 ±} 3.68(19.20) R					
Season						
Pre-monsoon (n=76)	29.39 ±} 1.92 T	76	0.633	0.15 (-.46-1.7)	18.27	<0.001*
	72.44 ±} 9.95 RH					
	8.28 ±} 12.15(1.90)R					
Monsoon (n=427)	29.15 ±} 1.40 T	427	1.35	0.12 (.32-2.38)	118.28	<0.001*
	85.25 ±} 6.77 RH					
	13.25 ±} 17.45(6.00)R					
Post-monsoon (n=75)	27.09 ±} 2.21 T	75	1.18	0.22 (.07-2.27)	28.49	<0.001*
	86.89 ±} 6.53 RH					
	20.60 ±} 29.79(5.20)R					
Winter (n=11)	22.09 ±} 3.53 T	11	Reference category			
	77.27 ±} 10.82 RH					
	4.98 ±} 11.02(1.70)R					

RH, relative humidity; R, rain fall; T, temperature;

* statistically significant

#### V. cholerae infection with no rainfall:

**Fig. 2:**
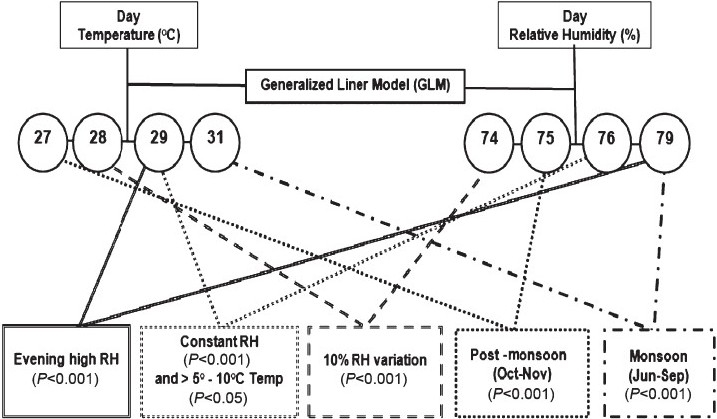
GLM identified *V. cholerae* favourable difference ranges of climatic factors with no rainfall (n=637).

The evening high RH favoured (*P*<0.001) *V. cholerae* infection when RH and temperature were 79 per cent and 29°C, respectively (Fig. 2). Zero difference relative humidity (*P*<0.001) co-incidence with moderate variation of day temperature (>5°C-10°C) that favoured *V. cholerae* infection when the RH and temperature were 76 per cent and 29°C, respectively. RH variation to 10 per cent in day time favoured (*P*<0.001) *V. cholerae* infection even when the temperature was 27°C. The monsoon appeared to be highly favourable for *V. cholerae* infection (*P*<0.001) that coincided with day time high humidity (79%) and high temperature (31°C). In addition, the post-monsoon season also showed significant association (*P*<0.001) with 27°C temperature and 75 per cent RH. None of the other factors had significant association with the categorized RH, temperature and seasons in both categories (Table III).

**Table III:** Generalized linear model (GLM) explored the *V.cholerae* infection with no rain fall events (n=637) associated with interval rang of relative humidity and temperature

R.H. Difference (Morning-Evening)	Mean ± SD	*V. cholerae* (n=637)	Parameter value	SE 95% likelihood CI	Wald Chi-square	*P* value
Evening high RH (n=132)	79.17 ± 8.98 RH	132	1.182	0.26 (-.43-2.79)	20.33	<0.001*
	29.28 ± 2.78 T					
Day’s equal RH (n=22)	76.18 ± 6.94RH	22	0.750	0.40 (-.97-2.47)	3.55	<0.001*
	29.29 ± 2.83 T					
≤10% (n=132)	73.63 ± 6.90RH	270	0.976	0.24 (-.62-2.57)	16.23	<0.001*
	28.41 ± 3.69 T					
>10-20% (n=132)	71.87 ± 6.89RH	132	0.856	0.254 (-.75-2.47)	11.39	0.001*
	27.84 ± 3.98 T					
>20-30% (n-60)	65.82 ± 8.78RH	60	0.233	0.25 (-1.40-1.87)	0.88	0.349
	27.33 ± 4.62 T					
>30-40% (n=17)	65.21 ± 4.27RH	17	0.338	0.27 (-1.42-2.10)	1.53	0.217
	29.96 ± 2.07 T					
>40% (n=4)	60.62 ± 2.95RH	4	Reference category			
	29.35 ± 1.46 T					
Temperature difference						
≤5° C (n=17)	82.62 ± 4.19RH	17	0.118	0.90 (-1.65-1.88)	0.017	0.897
	29.28 ± 2.14T					
<5-10°C (n=419)	75.69 ± 7.47RH	419	0.325	0.87 (-1.27-1.92)	0.139	0.709
	29.69 ± 2.59T					
<10-15°C (n=197)	68.10 ± 7.91RH	197	0.239	0.87 (-1.84-1.37)	0.075	0.784
	25.84 ± 4.22T					
<15°C (n=4)	65.00 ± 12.36RH	4	Reference category			
Season						
Pre-monsoon (n=200)	68.90 ± 8.23RH	200	0.352	0.13 (-.07-.77)	7.62	0.006*
	30.75 ± 2.09T					
Monsoon (n=151)	79.10 ± 6.31RH	151	0.769	0.19 (.31-1.23)	16.47	<0.001*
	30.86 ± 1.34T					
Post-monsoon (n=211)	74.72 ± 7.31RH	211	1.09	0.17 (.67-1.51)	39.54	<0.001*
	26.95 ± 2.30T					
Winter (n=75)	70.72 ± 8.30RH	75	Reference category			
	21.67 ± 2.38T					

RH, relative humidity; T, temperature;

* statistically significant

#### Unusual rise of V. cholerae infection in December 2008:

**Fig. 3:**
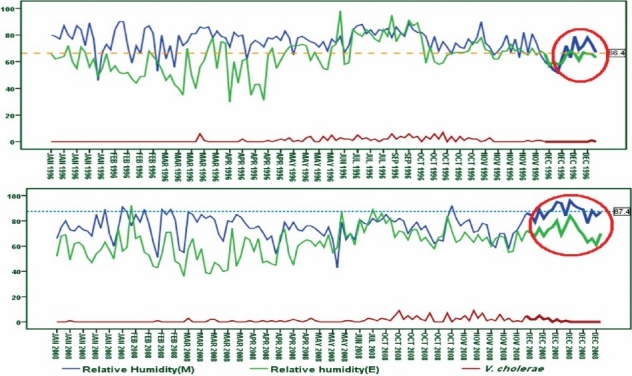
Depicting 21 per cent RH rise between 1996 and 2008 on unusual observed *V. cholerae* infection with no rain fall events.

The mean days RH (morning) has drastically increased to 21 per cent in December 2008 (87.44 ± 5.03) compared to December 1996 (66.43 ± 8.5) while the temperature remained constant. Previously, it was predicted that winter would be warmer than normal owing to the La Niña event in 2007-2008, which was the strongest since 1988-1989. This may be attributable to the sudden increase of RH as well as the *V. cholerae* infection (Fig. 3).

## Discussion

Diarrhoea remains the second most leading cause of death in children under five years of age^22^. In 2008, 9 million under 5 yr children died and 40 per cent of them were due to two diseases, pneumonia and diarrhea^23^. The estimated diarrhoeal disease burden from water, sanitation, and hygiene at the global level has revealed 4.0 per cent of all deaths and 5.7 per cent of the total diseases burden^24^.

The environmental factors influence the impact of the infectious diseases specially with season and space. The environment factors from ecosystem include physical, chemical and biological factors. Spread of cholera in West Bengal was related to its ecosystem, which favours *V. cholera*^6^. In addition to this, drainage, human behaviour, social customs and economic status were also related to the spread and persistence of the diarrhoeal disease^25^.

During early 1920s no positive correlation was found between cholera and rainfall in west Bengal^7^, as rainfall alone was not a contributory factor. The present study indicated a strong correlation between rainfall and incidence of cholera. Surface outwash, defective drainage and contamination of drinking water are the other important sources for cholera infection.

Climate is an important factor for *V. cholerae* persistence and spread in Bengal. Russel studies corroborated the declined death rate against decrease in the relative humidity in the some districts of Burdwan and Malda^26^. The other contributory factors for the increased infection due to *V. cholerae* are rainfall and RH. Hot and moist climate of the Bengal basin affects the general health of the people^27^. The relationship of cholera incidence with temperature and rainfall has been established in this study with statistical model approaches.

Russel and Sundararajan^28^ established that high temperature, correspondingly high RH and intermittent rainfall acted as ideal climatic conditions for the incidence of cholera. The present scenario of cholera in Kolkata revealed that ideal climatic condition depends upon the seasonality. In this study it was found that the temperature was neither low nor high but the humidity was high during rainfall. During this period, the zero difference day temperature highly favoured survival of *V. cholerae* along with the gradual increase in RH throughout the day with controlled temperature. During monsoon, RH was the maximum followed during post-monsoon. High RH was found to be associated with rainfall days as well as no rainfall days when the temperature was at moderate.

The observed RH, temperature and rainfall changes over thirteen year period were not constant owing to either El Niño or La Niño. The RH remained almost constant during raining season but minimized considerably when there was no rainfall. The progressive nature of *V. cholerae* infection was correlated with high RH both during raining and non-raining periods. Interestingly, about 21 per cent increase in RH and 1°C temperature during December 2008 was correlated with unusual increase in *V. cholerae* infection compared to December 1996. This finding demonstrated that the climate and health relation at Kolkata and the relative humidity plays a big role in the increased *Vibrio*-mediated infections.

Considering the infection of cholera in the past and its existing trends, it appears that the infection is related with multiple factors. To control epidemics of cholera, a symptomatic approach has to be made considering the clinical and environmental data. As shown in this study and in previous findings an inter-relation between temperature and relative humidity prevails in Kolkata with high RH during rainy season^29^,^30^.

### 

#### Time series analysis in diseases surveillance:

SARIMA model of infectious diseases surveillance is useful for forecasting the outbreaks^31^. In a 13 years data series, gradual changes in the cholera infection shifting was observed during the first four years (1996-99) with single peak, followed by gradual change into two peaks in a year. The scenario of cholera in Kolkata showed an initial increase from April and peaks in September- October which continued till December. From 1996-1999, cholera infection commenced in April and continued till December with 1 ^st^ peak in June and the 2^nd^ during September-October months. However, data collected during 2000 to 2006 showed that the cholera shifted 1 ^st^ peak from April to July and 2^nd^ peak from August to October and remained less in November 2008, which was correlated due to high RH. A WHO report estimates that 94 and 40 per cent of deaths were due to diarrhoeal diseases and malaria, respectively that has relation with environmental factors^32^.

In conclusion, our findings showed that *V. cholerae* infections increased due to rainfall events with high RH. In addition, the RH also favoured the *V. cholerae* infection during non-rainy season. The SARIMA model (1,0,0) (0,1,1) identified linear relationship (Positive relation) between *V. cholerae* infection and RH and increase of 10 per cent RH and 2°C temperature difference between rain and no rain fall occurrence. RH has favoured *V. cholerae* infection irrespective of the season. Zero variation in daily RH and median temperature propagate the *V. cholerae* infection. Persistent *V. cholerae* infection seems to be supported by overall higher RH (>80%) with moderate temperature (~29°C) and intermittent rainfall (~10 cm). While heavy rainfall supported the *V. cholerae* infection, high temperatures did not favour infection as shown in this statistical model.
